# A Review of Low-Frequency EPR Technology for the Measurement of Brain pO2 and Oxidative Stress

**DOI:** 10.1007/s00723-021-01384-5

**Published:** 2021-07-16

**Authors:** John Weaver, Ke Jian Liu

**Affiliations:** Department of Pharmaceutical Sciences, College of Pharmacy, University of New Mexico Health Sciences Center, Albuquerque, NM 87131

**Keywords:** *In vivo* EPR, brain pO_2_, oxidative stress

## Abstract

EPR can uniquely measure paramagnetic species. Although commercial EPR was introduced in 1950s, the early studies were mostly restricted to chemicals in solution or cellular experiments using X-band EPR equipment. Due to its limited penetration (<1 mm), experiments with living animals were almost impossible. To overcome these difficulties, Swartz group, along with several other leaders in field, pioneered the technology of low frequency EPR (e.g., L-band, 1–2 GHz). The development of low frequency EPR and the associated probes have dramatically expanded the application of EPR technology into the biomedical research field, providing answers to important scientific questions by measuring specific parameters that are impossible or very difficult to obtain by other approaches. In this review, which is aimed at highlighting the seminal contribution from Swartz group over the last several decades, we will focus on the development of EPR technology that was designed to deal with the potential challenges arising from conducting EPR spectroscopy in living animals. The second half of the review will be concentrated on the application of low frequency EPR in measuring cerebral tissue pO_2_ changes and oxidative stress in various physiological and pathophysiological conditions in the brain of animal disease models.

EPR can uniquely measure paramagnetic species, i.e., compounds with an unpaired electron. Although commercial EPR was introduced in 1950s, the early studies were mostly restricted to chemicals in solution or cellular experiments using X-band EPR equipment. Due to its limited penetration (<1 mm), experiments with living animals were almost impossible. Furthermore, many paramagnetic species are short lived, preventing them from accumulating to higher concentration, which further limits the application of EPR in biomedical research. To overcome these difficulties, Swartz group, along with several other groups, including the Zweier [1], Berliner [2], Eaton [3] and Halpern [4] Labs, pioneered the technology of low frequency EPR, such as L-band (up to 1–2 GHz). The development of low frequency EPR and the associated probes have dramatically expanded the application of EPR technology into the biomedical research field, providing answers to important scientific questions by measuring specific parameters that are impossible or very difficult by other approaches. In the following, we will focus on the development of EPR technology for *in vivo* measurement of tissue pO_2_ and oxidative stress, highlighting the seminal contribution from Swartz group.

## 1. Development of EPR technology for *in vivo* measurement of tissue pO_2_ and oxidative stress

### 1.1. Development of low frequency EPR instrument

*In vivo* EPR with direct detection from living animal potentially can provide information about biological processes in which tissue oxygenation or free radicals play critical important roles. The advantage of direct *in vivo* EPR measurement in living animals is obvious, as the measurement is the most physiologically relevant and the measurement is in real time at the location of interest.

**Figure 1.**
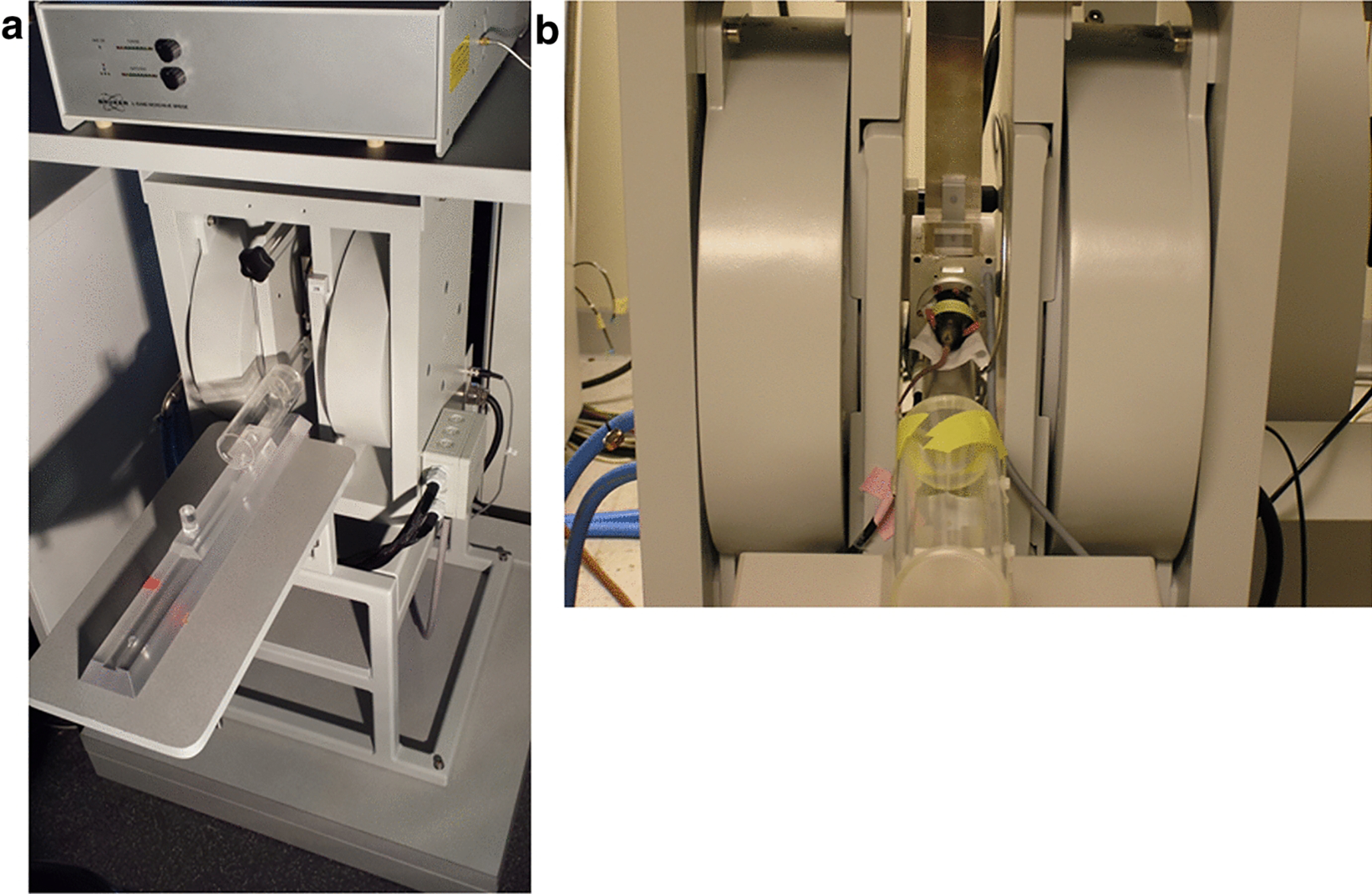
Low frequency (L-band) EPR spectrometer and representative animal placement in the instrument. **a** Bruker Biospin (Billerica, MA) L-band (*in vivo*) EPR spectrometer and imager with magnetic field gradient assembly. **b** Animal placement inside an *in vivo* EPR spectrometer for EPR imaging, spectroscopy and oximetry measurements.

Conventional EPR spectrometers operate at about 9 GHz (X-band). While these spectrometers offer excellent detection sensitivity, they have a very low penetration range of 1 mm due to non-resonance absorption of incident microwave energy by high content water in living animals. In order to overcome these difficulties and increase penetration range, Swartz and colleagues lead the field in developing the low frequency EPR instrument together with the associated probes for making measurements of important physiological parameters. The majority of low frequency EPR spectrometers operate at a frequency of ~1 GHz, which provides a good compromise between detection sensitivity and depth of microwave penetration. A few laboratories have successfully used a spectrometer operating around 250 MHz for EPR spectroscopy and imaging experiments in small animals [4]. A highly innovative concept was the development of the surface probe resonator [5, 6] which can be placed anywhere against the living subject, allowing ready localization of the EPR signal from the region of interest. This concept is a revolutionary departure from the traditional design of the EPR resonator, where the subject is placed inside the resonator. The development of surface detectors and insertable detectors have very significantly extended the types of experiments that can be carried out in large complex system such as perfused organs and intact, living animals [7]. Following their initial success, they were able to design newer generation of surface-coil resonators that have further improved detection sensitivity and automatic frequency matching system [8–11]. In recent years, they further developed an EPR spectrometer that was specially designed and constructed for EPR spectroscopy in humans [12]. These achievements in *in vivo* EPR instrumentation development greatly expanded the application of EPR in biomedical research (Fig. 1). More importantly, the biologically important parameters measured by EPR are often not obtainable by other methods.

### 1.2. Development of EPR oximetry probes

Oxygen (O_2_) plays such an important part in life, and surprisingly measuring O_2_ level in living subjects accurately, such as human and animals, has been challenging. Being paramagnetic itself, O_2_ can interact with EPR detectable species to cause linewidth alterations that can be calibrated to precisely measure the O_2_ level in the surrounding tissue. However, in order for the oximetry probes to be useful, they need to be: a) inert, causing minimal toxicity and immune response; b) sensitive, providing relatively large dynamic range of linewidth changes in response to O_2_ changes; and c) stable, allowing repetitive measurements over days or weeks for longitudinal studies. In the early days of EPR oximetry, nitroxides were the common probes for measuring intracellular and/or extracellular oxygen concentration [13–16]. However, the sensitivity of nitroxide to oxygen changes is low, and nitroxides can undergo bioreduction to EPR-inactive species readily. The discovery of stable EPR oximetry probes, particularly the solid-state particulate materials, dramatically expanded the capability of EPR oximetry in measuring tissue oxygenation in living animals. Over the last several decades, a series of probes have been developed in various labs, with significant contribution from Swartz lab.

**Figure 2.**
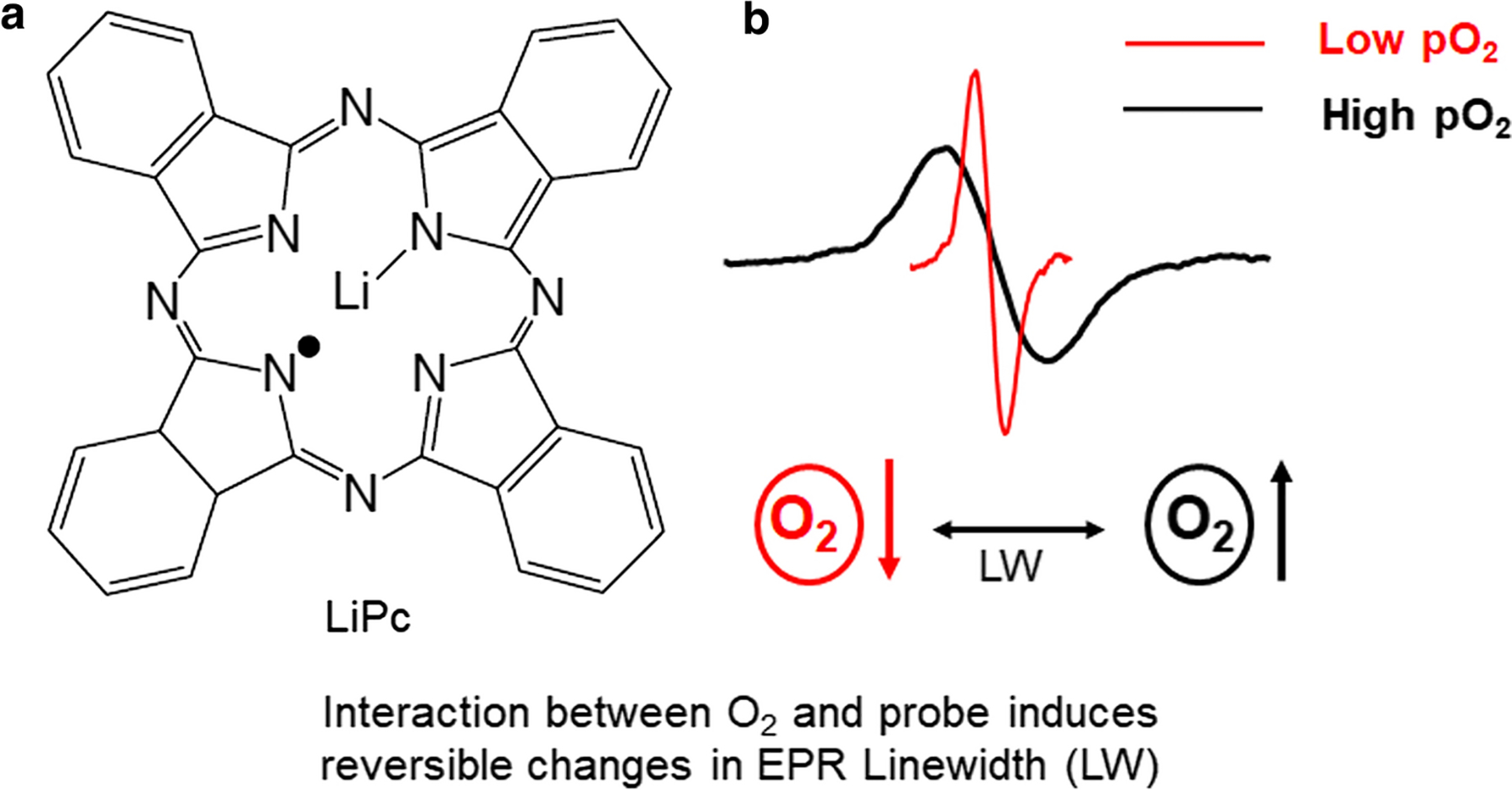
Lithium phthalocyanine (LiPc) EPR oximetry spin probe and variations of LiPc EPR linewidth with O_2_ concentration. **a** Structure of LiPc EPR oximetry spin probe. **b** Representative narrow EPR linewidth (red) in the presence of low O_2_ concentration and broader EPR linewidth (black) in the presence of higher O_2_ concentration.

Lithium phthalocyanine (LiPc) was the first of series of particulate oximetry probe. LiPc is a prototype of a generation of synthetic, metallic-organic, paramagnetic crystallites that appear very useful for *in vitro* and *in vivo* EPR oximetry (Fig. 2). The peak-to-peak line width of the EPR spectrum of LiPc is a linear function of the partial pressure of oxygen (pO_2_); this linear relation is independent of the medium surrounding the LiPc. It has an extremely exchange-narrowed spectrum (peak-to-peak line width = 14 mG in the absence of O_2_). Physicochemically, LiPc is very stable; its response to pO_2_ does not change with conditions and environments (e.g., pH, temperature, redox conditions) likely to occur in viable biological systems. These characteristics provide the sensitivity, accuracy, and range to measure physiologically and pathologically pertinent O_2_ tensions (0.1–50 mmHg; 1 mmHg = 133 Pa). The application of LiPc in biological systems has been demonstrated in measurements of pO_2_ *in vivo* in the heart, brain, and kidney of rodents [17–20].

Fusinite is one type of coal maceral that occurs with various abundance in the seams of coal mines. It is insoluble, amorphous, aromatic, and of organic origin. The peak-to-peak line width of the first derivative EPR spectrum of fusinite is reversibly broadened by O_2_. The extent of broadening per unit of pO_2_ is unusually large, exceeding that of nitroxides by almost two orders of magnitude. This paramagnetic property of fusinite, combined with its very stable physicochemical properties and low toxicity, was shown to be of utility in the measurement of pO_2_ *in vitro* and *in vivo*. Fusinite particles are endocytosed by cells *in vitro*; this is useful for intracellular O_2_ measurements with commercially available EPR spectrometers operating at 9.1–9.3 GHz. For measurement of O_2_ *in vivo* using low frequency EPR, fusinite provides a sensitive and persistent means to measure pO_2_ in tissues. Particles implanted into the gastrocnemius muscle of A/J mice remained interstitially in the same position for months with undiminished sensitivity to pO_2_ and no specific toxic effects [21, 22]. A similar material, a type of Welsh coal, gloxy, was found to have valuable EPR features that allow accurate measurement of low O_2_ tensions *in vivo*; these include large O_2_-dependent changes in linewidth, a high number of paramagnetic spin centers, and stability in tissue allowing repeated pO_2_ measurements to be made *in vivo* with high precision [23].

India ink shares many of the favorable properties of LiPc and fusinite coal, but it already is in widespread use in humans. In addition to being used for writing and painting, in many parts of the world, it is also used for decoration as tattoos. In clinical medical practice, it has been already been used extensively in patients as a short term and permanent marker in the skin mucosal tissue, and tumors. The first EPR measurements in a human was made using the India ink in a pre-existing tattoo. The EPR spectra of India ink are very sensitive to the pO_2_, thereby making it feasible to use this approach to measure pO_2_ in tissues in patients. This provides a means to measure this parameter directly with a sensitivity, accuracy, and repeatability that had not been available previously, and thereby able to individualize and guide treatment of diseases such as cancer and peripheral vascular insufficiency [24–27]. Indeed, *in vivo* measurements in human subjects were made using low frequency EPR spectroscopy and surface loop resonators, which enable measurements to be made at superficial sites through a non-invasive (after placing the ink in the tissues) and repeatable measurement procedure. EPR oximetry studies in human subjects included measurement of subcutaneous pO_2_ in the feet of healthy volunteers to develop procedures that could be used in the treatment of peripheral vascular disease and to follow tumor oximetry during courses of radiation and chemotherapy to optimize O_2_-dependent therapies. These studies demonstrate the feasibility of EPR oximetry in a clinical setting and the potential for more widespread use in the treatment of these and other O_2_-dependent diseases [27].

### 1.3. Measurement of reactive oxygen/nitrogen species (ROS/RNS) in intact animals using low frequency EPR

Generation of ROS, and the resulting oxidative stress, have been implicated in a variety of disease, from cancer to cardiovascular disease and diabetes. Measuring the oxidative stress condition and the specific reactive species, has been challenging, because they usually are short lived and/or do not accumulate to high enough concentration to be detectable. It is even more difficult if the measurement is desired in an animal model. Many different EPR-based approaches have been development, each with its own strengths and weaknesses. Utilizing the unique capability of L-band EPR spectrometer in detecting free radicals *in vivo* in combination with the commercially available spin probes, Swartz group and others published a series of papers demonstrating the *in vivo* and *ex vivo* generation of free radicals in a variety of conditions [2, 29–39].

Since free radicals in general are short lived and/or exist in very low concentrations in biological systems, the technique of spin trapping is often used, which converts the short lived radicals into longer lived species, the “spin adduct,” detectable by EPR. In order to determine the optimal spin traps for each specific study, a variety of spin traps with diverse properties were evaluated [33, 34]. It was shown that for a successful *in vivo* spin trapping experiment, the stability of the spin trap is not of major concern, but the time course of distribution may be important [33]. Using a low frequency EPR spectrometer, the spin trapped SO_3_•- radical was observed both with DMPO and DEPMPO directly in the intact mouse. DEPMPO was found to be a good candidate for trapping radicals in functioning biological systems and represents an improvement over the commonly used trap DMPO [35, 36]. Using the similar approach, the first direct *in vivo* EPR detection of hydroxyl radical adducts in intact mice was demonstrated with DEPMPO [37].

*In vivo* EPR spectroscopy was also applied to measure nitric oxide (NO•) and pO_2_ directly, and non-invasively, from tissue. Diethyldithiocarbamate (DETC) was used as the spin trap to complex with NO• in the tissue, while Gloxy (an oxygen-sensitive, paramagnetic material) was also implanted into the tissue of interest (brain or liver). These studies demonstrate the potential usefulness of this technique for making direct *in vivo* measurements of NO• and pO_2_ simultaneously from tissue in living animals [38, 39]. These studies demonstrate the powerful combination of L-band EPR with spin trapping in detecting the generation of reactive species in intact animal models.

### 1.4. Short summary outlining the rest of the review

The text above described the development of the low frequency EPR spectrometer and detectors, which are designed to deal with the potential problems arising from trying to conduct EPR spectroscopy in a living animal with consequent large amounts of lossy materials and a variety of movements from physiological processes. Despite limitations and technical challenges, the resulting L-band EPR has a consequent depth of sensitivity of about 10 mm, which is adequate for most rodent studies. Combining the increased detection range with the increasingly available diverse probes, low frequency EPR has become a powerful tool to investigate the role of pO_2_ and oxidative stress in the development of different diseases [40, 41]. Unquestionably, we direct readers to explore increasing contributions and significant developments in EPR technology and methodology in an expanding field larger than and beyond the scope of this manuscript. However, the second half of this review will be focused on the application of low frequency EPR in measuring cerebral pO_2_ changes and oxidative stress in various physiological and pathophysiological conditions in the brain, highlighting particular contributions from Swartz group.

## 2. EPR Applications in Brain Research

### 2.1 Cerebral pO_2_ under different anesthetics and changes in respired O_2_

Adequate cerebral pO_2_ and redox balance is critical to brain function and monitoring cerebral O_2_ together with oxidative stress levels, particularly *in vivo* and in real time, is of high significance to understanding brain physiology. Early studies by the Swartz group focused on establishing low frequency EPR oximetry as a unique method to determine cerebral pO_2,_ *in vivo* and in real time, and provided proof-of-concept evidence of minimally invasive, sensitive, accurate, and reproducible localized cerebral pO_2_ measurements using LiPc, a particulate paramagnetic O_2_ sensitive probe [18, 42]. Far from the comprehensive list in literature, the Swartz group complimented their initial findings with ongoing studies and continued innovations in EPR methodology, instrumentation and technology to study tissue pO_2,_ particularly in the brain [10, 19, 43–50]. These studies demonstrated and verified LiPc linewidth conversions and signal height responses correlated to cerebral pO_2_ changes, *in vivo*. Different classes of anesthetics, including ketamine-xylazine, isoflurane and halothane, and accurate determinations of cerebral pO_2_ changes from varying amounts of O_2_ concentration in breathing gas or during hypobaric hypoxia models in anesthetized and awake animals were studied. The reports also vigorously examined the electrochemical, magnetic and physicochemical properties of LiPc, and established its use for highly accurate EPR oximetry measurements over both short and long periods of time (*e.g*., days and weeks), particularly in the brain. Thus, the Swartz group set the stage for EPR as a tool for monitoring, prognosis and analysis of cerebral pO_2_ under various brain conditions in animal models, leading and guiding potential treatments of disorders and diseases.

### 2.1 Cerebral pO_2_ and Oxidative Stress in Hypertension and Drug Abuse Models

Cerebral pO_2_ related to vascular cognitive impairment (VCI), and cognitive impairment and dementia (VCID)/Alzheimer’s Disease (AD) progression had not been thoroughly explored, although hypoxia is considered a major vascular contributor. The deep white matter (WM) is a common site of hypoxic/ischemic injury in the elderly where it is associated with cognitive decline, gait disturbances, and focal ischemia leading to VCI. Hypertension is also a VCI contributor leading to reduced cerebral blood flow and hypoxia and can accelerate AD progression. Modernization and optimization of EPR instrumentation and methodology for *in vivo* and in real time brain oximetry provided a unique and direct *in vivo* method for evaluating cerebral pO_2_ in hypertension, VCI, and VCID/AD animal models. LiPc chronically implanted in the WM and dorsolateral cortex gray matter of spontaneously hypertensive-stroke prone (SHR/SP) rats evaluated changes in and significance of cerebral pO_2_ and hypertensive damage in longitudinal VCI and VCID AD model studies, respectively [51, 52]. A continued decrease in WM pO_2_ was associated with vascular damage in the VCI model, and attenuation in gray matter pO_2_ suggested hypoxic hypoperfusion initiates a neuropathological cascade involving free radical production, microglial activation, blood-brain barrier (BBB) disruption and tau hyperphosphorylation in the VCID AD model.

In a separate series of SHR/SP rat studies, Lee and colleagues used EPR techniques developed by their group, a novel BBB-permeable redox sensitive EPR spin probe [53], and EPR imaging advancements by Zweier, Kuppusamy and others [54–56] to evaluate oxidative stress in the SHR/SP rat brain. While the exact brain region could not be determined since EPR imaging, alone, does not provide anatomical information, an increase in oxidative stress was observed compared to Wistar-Kyoto controls. It was also suggested EPR could, theoretically, assess and screen drugs for antioxidant effects on oxidative stress in these models. EPR imaging clearly visualized accelerated changes in brain redox status and oxidative stress in other transgenic AD mice models and reported AD treatments or drugs improved this unbalanced redox state and potentially prevented AD impairments [57–60]. Together, these studies highlighted utilizing EPR to yield novel information regarding the importance of cerebral pO_2_, oxidative stress and redox status in the pathophysiology of hypertension, VCI and AD, including quantitative evaluation of potential treatments, which would not otherwise be elucidated in such a rigorous fashion.

**Figure 3.**
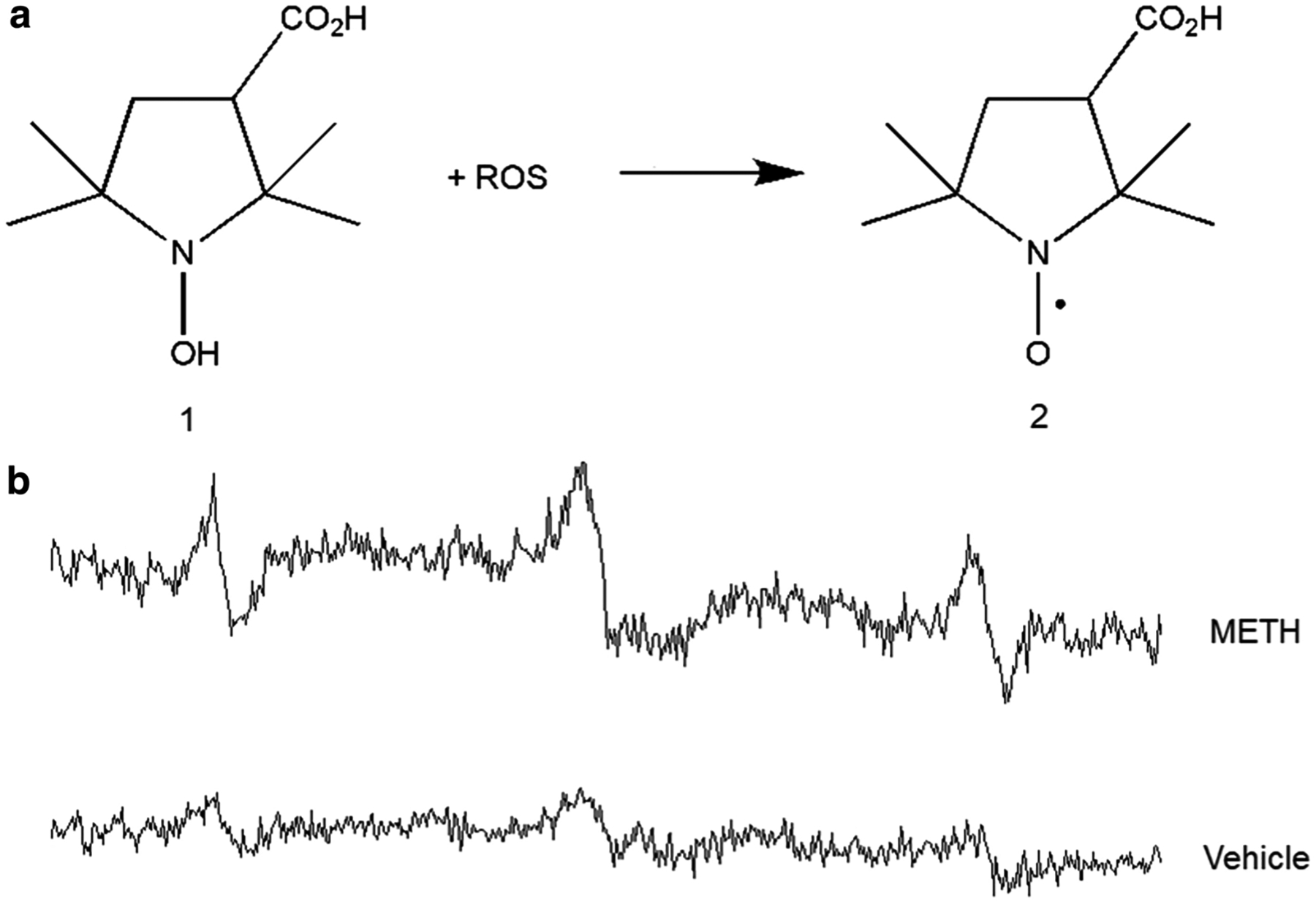
EPR measurement of oxidative stress in the brain. **a** Reaction mechanism of EPR-silent hydroxylamine CP-H (1-hydroxyl-3-carboxy-2,2,5,5-tetramethylpyrrolidine) [1] to the EPR-active nitroxyl radical [2] as an indicator for oxidative stress. **b** EPR spectra of [2] in the brain of a mouse subject to 3 consecutive days of acute methamphetamine (METH, 8 mg/kg iv per day) challenge vs vehicle (0.9% saline). CP-H [1] (1 mmole/kg, i.p.) was injected 5 min before METH. Increased EPR signal of [2] was recorded 60 min after METH indicating reactive oxygen species (ROS) generation (unpublished work).

Similarly, EPR oximetry and imaging has been used to monitor cerebral pO_2_ and redox status in drug challenge, abuse and neurotoxicity animal models. Utilizing *in vivo* EPR-LiPc oximetry, a significant attenuation in striatal pO_2_ was observed following acute methamphetamine (METH) exposure which did not fully recover 24 hours after METH challenge and was sustained with consecutive daily METH administration [61]. In a separate study, brain redox status was evaluated in mice after repeated METH challenge using an EPR imaging and a redox-sensitive BBB-permeable spin probe [62]. A significant shift in redox status was visualized which returned to nearly control levels 2 weeks after METH withdrawal. Unpublished work from our lab (Fig. 3) also investigated the possibility of using EPR spectroscopy and ROS-sensitive spin probes to study oxidative stress, *in vivo*, after acute METH challenge. Increased EPR signal suggested ROS generation after METH challenge. Again, a direct correlation between the site of cerebral pO_2_ attenuation and redox status shift cannot be made; nonetheless, it is important to emphasize that oxidative damage can be proportional to the amount of O_2_ present and/or mediated through cell signaling that occurs at critical O_2_ levels. Thus, EPR, coupled with novel oximetry and spin probes, may provide a unique means to better understanding cerebral O_2_ and brain redox status in drug abuse and challenge models if direct measurements can be obtained and associated with other processes at the site of interest [43].

### 2.3 Free Radicals in the Brain After Onset of Seizures and Traumatic Brain Injury

Free radical levels, such as NO•, may influence brain function, serving as a signal molecule, neuroprotector or as a neurotoxin and mediator of inflammation. Several groups, including the Swartz lab, demonstrated early developments in *in vivo* and *ex vivo* EPR spectroscopy and spin trapping to assess brain generated free radicals such as NO• in animal models [2, 29–39] alongside evidence of increases in NO• putatively dependent on inducible nitric oxide synthase (iNOS) [63]. These studies paved the way for using EPR to reinforce a role for free radicals in traumatic brain injury (TBI) and seizure animal models.

In TBI model studies, NO• was assessed, *ex vivo*, in brain tissue using a lipophilic iron spin trap, demonstrating changes in NO• after TBI appeared dependent on the animal developmental stage [64]. Increases in NO• in the neonatal brain was starkly different than the significant decrease in NO• (even below control values) in the mature brain. Several hypotheses were offered for the increase in younger brains, and NO• decrease in mature animals was suggested via NO• reaction with other RNS, potentially influencing oxidative damage. In a separate study, another group would demonstrate a 2-fold greater EPR spin trapped-NO signal in wild-type versus iNOS knock-out (KO) mice after TBI in addition to evaluating ascorbate levels and oxidative stress markers by other methods. Diminished NO• and greater ascorbate loss in iNOS KO mice suggested a role for iNOS-derived NO• as an endogenous antioxidant after TBI when iNOS induction is maximal [65]. Of note, a human tissue research study by the same group found increased oxidative stress markers, reduced total antioxidant reserve, and a significant increase in EPR detectable ascorbate radical with ascorbate reduction, likely associated with its free radical oxidation [66]. Furthermore, similar increases in oxidative stress biomarkers, reduction in antioxidant reserve, and increases in EPR detectable ascorbate and hydroxyl radicals were observed in TBI rat models [67, 68]. Induction of iNOS in injured brains of TBI patients would also support a crucial role for iNOS, NO• and free radicals in cerebrovascular damage and/or secondary brain damage following TBI [69].

Similarly, several studies have utilized *in vivo* and *ex vivo* EPR spectroscopy and imaging to monitor free radicals, oxidative stress and antioxidant efficiency in seizure animal models. Significant increases in spin trapped-NO and hydroxyl radicals were observed in different brain regions at peak-time and after induction of seizure [70–72]. EPR imaging of seizure-induced animals also revealed a decrease in glutathione potentially leading to increased oxidative stress [73, 74]. One group performed a series of studies using EPR and novel spin probes to measure generation of free radicals, lipid radicals, oxidative stress, redox state and antioxidant efficacy in varying brain regions, suggesting increases in free and lipid radicals contribute to a collapse of the redox state in seizure-induced injury [75–78]. Together, these EPR studies confirmed the importance of monitoring free radical levels, oxidative stress and redox status in TBI and seizures, strengthening and defining a role of free radicals and oxidative stress in brain injury and damage, which could help the development of novel therapies.

### 2.4 Cerebral pO_2_ and Oxidative Stress in Ischemic Stroke Models

Considering the vulnerability of the brain to hypoxic/ischemic injury, EPR oximetry, spectroscopy and imaging have become important tools for the study of stroke pathophysiology since cerebral pO_2_ and free radical generation, metabolism will vary dramatically over the brain tissue structure during and after ischemic stroke [79–81]. Additionally, to develop effective stroke treatments, it is crucial to understand the effect of potential therapies on cerebral pO_2_ and physiological processes in the core, penumbra and contralateral regions of the ischemic brain. While there have been countless ischemic stroke animal model studies over the last several decades, we will highlight select literature in the stroke field by the Swartz group and others using *in vivo* and *ex vivo* EPR methodology.

Building upon the unique capability of *in vivo* single-site EPR oximetry, Swartz and colleagues would develop bilateral or multi-site implantation of LiPc for simultaneous localized measurements of interstitial pO_2_ at multiple sites in the same animal [82,83]. Accordingly, analysis of both absolute values and temporal cerebral pO_2_ changes in the penumbra, core and/or contralateral regions, sometimes simultaneously, has been achieved during acute ischemia and reperfusion (IR) [42, 79, 83–88]. The Swartz group would also pioneer implantable resonators with multiple sensors at variable lengths and capable of deeper tissue measurements to study spatially distributed temporal cerebral pO_2_ changes in ischemic animal models [80, 89–91]. With these advancements and innovations, EPR oximetry would prove to be a useful tool to investigate the effect of IR on the cerebral pO_2_ at several sites, simultaneously, and repeatedly over hours and even days. A synopsis of each individual study is beyond the scope of this review, but altogether, reports would show varying localized effects of IR on cerebral pO_2_, mostly due to stroke and/or reperfusion duration and differences in O_2_ consumption rates, microcirculation and metabolism between the brain regions.

These single and multi-localized site cerebral pO_2_ measurements by the Swartz group and others have been complimented by advancements in EPR imaging with optimal imaging agents or spin probes for mapping cerebral pO_2_, oxidative stress and redox status over larger brain regions. The lipophilic, pharmacokinetic and pharmacodynamic properties of soluble O_2_-sensitive BBB permeable labile-ester-nitroxides have been vigorously studied [92,93], and mapping spatially distributed cerebral pO_2_ using labile-ester-nitroxides further demonstrated heterogeneous changes throughout the brain tissue after cerebral ischemia [79, 95]. Additionally, efforts to improve the methodology with isotopically (^2^H,^15^N)-substituted nitroxides, proven to be ~2.5-fold more pO_2_ sensitive than their ^1^H,^14^N counterparts have shown promise for future oximetry studies [32, 81, 96–98].

EPR mapping of oxidative stress and/or redox status shifts after IR with BBB-permeable redox sensitive nitroxides or spin probes has been described by various groups [99–101]. Studies demonstrated increased oxidative stress during IR and supported findings from accompanying EPR spectroscopy IR studies using redox sensitive nitroxides and free radical spin trapping [79, 99–108]. Together, EPR imaging and spectroscopy investigations would provide evidence of a role of increased oxidative stress, lipid, NO• and hydroxyl radicals in a cascade of reactions leading to ischemic-hypoxic neuronal damage. Corresponding EPR studies also demonstrated neuroprotective effects of antioxidants on free radical formation in the ischemic brain [102, 109–112]. As mentioned earlier, the potential shortcoming of EPR imaging is the lack of anatomical information, but efforts are ongoing to improve methods for determination and/or co-registration of regional cerebral locations defined by EPR imaging [81, 98, 113].

EPR methodologies not only provide critical information on cerebral pO_2_ and shifts in redox status for analyzing stroke pathophysiology, but may potentially be novel tools for developing, improving and optimizing strategies for stroke treatment. To illustrate, several EPR oximetry reports by the Swartz group and others highlight the importance of monitoring cerebral pO_2_ during hyperbaric oxygen (HBO) and normobaric oxygen (NBO) treatment of acute ischemic stroke. NBO treatment applied during ischemia restores penumbra pO_2_ back to or above pre-ischemic levels [79, 84, 114] leading to neuroprotection, while NBO during reperfusion increases penumbra pO_2_ to twice pre-ischemic levels [84], offering no significant neuroprotection. Alternatively, HBO treatment during acute IR revealed no significant differences in cerebral pO_2_ compared to controls even though HBO during ischemia exhibited neuroprotective effects [87]. These studies emphasized experimental setup, timing and duration of HBO, NBO or any O_2_ therapy approach are possibly the most important factors responsible for effective neuroprotection without the potential of increased oxidative stress or damage, offsetting any neuroprotective properties [87, 115–117]. Ultimately, these studies highlight the importance of monitoring cerebral pO_2_ and redox status during acute IR and EPR as a useful tool to address fundamental questions regarding the effectiveness of stroke treatments or therapy in animal models.

## 3. Conclusion

Over the past several decades, Dr. Swartz and colleagues have trailblazed, influenced and made significant strides in the development and advancement of EPR technology, instrumentation and methodology. Herein, we reviewed the Swartz group impact on the EPR field, highlighting his lab’s significant contributions to the applicability of *in vivo* and *ex vivo* EPR in brain research. Specifically, the Swartz Lab modernized EPR sensitive spin probes, multi-site measurements, implantable resonators and instrument innovations to revolutionize biomedical, preclinical and clinical research. These distinguished scientific endeavors have fashioned EPR oximetry, spectroscopy and imaging as novel applications to evaluate, quantitate and define cerebral pO_2_, free radicals and redox status in brain pathophysiology under a variety of conditions, disorders and diseases, including but not limited to TBI, seizure, drug challenge, hypertension and ischemic stroke animal models. While the possibility of EPR utilization in clinical settings is challenged by EPR instrumentation, methodology and regulatory restrictions [27, 118–120], continuous efforts are ongoing to develop and translate the unique technology of EPR to clinical practice for prognosis, prediction, noninvasive diagnosis, and/or treatment analysis of brain conditions.
